# Protective Effects of Lemon Juice on Alcohol-Induced Liver Injury in Mice

**DOI:** 10.1155/2017/7463571

**Published:** 2017-04-16

**Authors:** Tong Zhou, Yu-Jie Zhang, Dong-Ping Xu, Fang Wang, Yue Zhou, Jie Zheng, Ya Li, Jiao-Jiao Zhang, Hua-Bin Li

**Affiliations:** ^1^Guangdong Provincial Key Laboratory of Food, Nutrition and Health, School of Public Health, Sun Yat-sen University, Guangzhou 510080, China; ^2^South China Sea Bioresource Exploitation and Utilization Collaborative Innovation Center, Sun Yat-sen University, Guangzhou 510006, China

## Abstract

Chronic excessive alcohol consumption (more than 40–80 g/day for males and more than 20–40 g/day for females) could induce serious liver injury. In this study, effects of lemon juice on chronic alcohol-induced liver injury in mice were evaluated. The serum biochemical profiles and hepatic lipid peroxidation levels, triacylglycerol (TG) contents, antioxidant enzyme activities, and histopathological changes were examined for evaluating the hepatoprotective effects of lemon juice in mice. In addition, the in vitro antioxidant capacities of lemon juice were determined. The results showed that lemon juice significantly inhibited alcohol-induced increase of alanine transaminase (ALT), aspartate transaminase (AST), hepatic TG, and lipid peroxidation levels in a dose-dependent manner. Histopathological changes induced by alcohol were also remarkably improved by lemon juice treatment. These findings suggest that lemon juice has protective effects on alcohol-induced liver injury in mice. The protective effects might be related to the antioxidant capacity of lemon juice because lemon juice showed in vitro antioxidant capacity.

## 1. Introduction

Alcohol abuse and alcoholism could lead to serious health and socioeconomic problems worldwide. Chronic excessive alcohol consumption (more than 40–80 g/day for males and more than 20–40 g/day for females) could lead to several illnesses, such as gastrointestinal damage, pancreatitis, alcoholic liver disease, neurologic disorders, diabetes mellitus, and cancer [1, 2]. Among these diseases, alcoholic liver disease has attracted more attention due to its high morbidity and mortality. Alcoholic liver disease is a major type of chronic liver disease throughout the world and can progress to liver cirrhosis and liver cancer.

Chronic alcohol consumption can generate abundant reactive oxygen species (ROS), including superoxide anion radical (O_2_^−•^), hydroxyl radical (OH^•^), and hydrogen peroxide (H_2_O_2_). The ROS can react with most cellular macromolecules and subsequently cause cellular damage [3]. Therefore, the excessive ROS induced by alcohol is regarded as an important factor in the development of alcohol-induced liver injury. Various enzymatic and nonenzymatic antioxidants are related to protecting cells against ROS. Antioxidant enzymes include catalase (CAT), superoxide dismutase (SOD), and glutathione peroxidase (GPx), and nonenzymatic antioxidants include glutathione (GSH), vitamin E, ascorbate, vitamin A, and ubiquinone [4]. Nonenzymatic antioxidants can be enhanced by antioxidant intake. In recent years, many natural products that have abundant antioxidants were reported to possess the effect of scavenging free radicals and protecting the liver from oxidative damage [4, 5].

Lemon is a popular fruit consumed as juice and contains high contents of vitamins and polyphenols (mainly flavonoids), such as hesperidin, eriocitrin, naringin, neohesperidin, rutin quercetin, chlorogenic acid, luteolin, and kaempferol [6]. The in vivo and in vitro experiments have shown that lemon has various health benefits, such as anticancer effect, antimicrobial effect, lipid-lowering effect, and protective effect against cardiovascular diseases [6]. In addition, lemon is used to treat liver ailments in tribal medicine [7]. However, effects of lemon juice on chronic alcohol-induced liver injury have not been reported in the literature. The objective of this study is to investigate the effects of lemon juice on chronic alcohol-induced liver injury in mice. In addition, the in vitro antioxidant capacities of lemon juice were evaluated. The results of this study could supply valuable information for the general public to reduce harm of alcohol consumption.

## 2. Materials and Methods

### 2.1. Chemicals and Reagents

The compounds 6-hydroxy-2,5,7,8-tetramethylchromane-2-carboxylic acid (Trolox), 2,2′-azinobis(3-ethylbenzothiazoline-6-sulfonic acid) diammonium salt (ABTS), 2,4,6-tri(2-pyridyl)- S-triazine (TPTZ), quercetin, gallic acid, and Folin–Ciocalteu's phenol reagent were purchased from Sigma-Aldrich (St. Louis, MO, USA). Assay kits for the determination of SOD, lipid peroxidation, CAT, and TG contents were purchased from Nanjing Jiancheng Bioengineering Institute (Nanjing, China). Other chemicals were of analytical grade.

### 2.2. Materials

Lemon was obtained from markets in Guangzhou, China. The fruit was cleaned with deionized water. The edible portion was weighed precisely and mixed with deionized water (1 : 1, m/v), and the mixture was ground into a homogenate with a homogenizer. Then, the homogenate was centrifuged at 5,000*g* for 10 min, and the supernatant was obtained. The supernatant was used for the measurement of antioxidant capacity, total phenolic contents (TPC), and total flavonoid contents (TFC) and for animal experiments. Moreover, in animal experiments, the original supernatant and the diluted supernatant (1 : 5 and 1 : 10, m/v) were used as the high, medium, and low dose, respectively. The lemon juice was freshly prepared before gavage every time.

### 2.3. Animal Study

Male C57BL/6 mice (20–25 g) were employed in this study. Thirty mice were randomly divided into 5 groups, each group containing 6 mice. They were maintained in a SPF laboratory animal room, which kept a 12 h light/dark cycle at 22 ± 0.5°C with 40%–60% relative humidity. The animal study was performed according to the “Principles of Laboratory Animal Care” and approved by the Institutional Animal Ethics Committee of Sun Yat-sen University. The model group was treated daily with ethanol and distilled water (10 mL/kg) at the same time; the lemon juice treatment groups were treated daily with different concentrations (high dose 1 : 1 (m/v), medium dose 1 : 5, and low dose 1 : 10) of lemon juice (10 mL/kg) and ethanol simultaneously; the control group was treated daily with isometric distilled water. The model group and the lemon juice treatment groups were given ethanol according to the following ways: 35% ethanol (v/v) at a dose of 3 g/kg body weight for 7 days, 40% ethanol (v/v) at a dose of 4 g/kg body weight for the next 7 days, and 52% ethanol (v/v) at a dose of 5 g/kg body weight on the 15th day [8]. All the intervention methods were intragastric administration. The blood and liver were collected from mice 9 h after the last ethanol administration. The blood sample was centrifuged at 4,000*g* for 10 min and the serum was collected. The obtained serums were stored at −22°C before determination. A piece of tissue was taken from liver and fixed in 4% paraformaldehyde, and then the remaining liver tissue was stored at −22°C until use.

### 2.4. Measurement of Biochemical Parameters in the Serum

The levels of ALT, AST, and TG in serum were determined by a Hitachi-7180 automated biochemistry analyzer (Hitachi, Japan) with the corresponding reagent kit.

### 2.5. Measurement of TG and Antioxidant Enzyme Activities in the Liver

The levels of TG, SOD, and CAT in liver tissue were measured using the commercial detection kits according to the manufacturer's instructions.

### 2.6. Measurement of Lipid Peroxidation Levels in the Liver

The levels of lipid peroxidation in liver tissue were measured by thiobarbituric acid (TBA) method using the commercial detection kits according to the manufacturer's instructions. The reference standard was malondialdehyde (MDA), and the results were expressed as nmol MDA equivalent/mg prot.

### 2.7. Liver Histopathological Assessment

The liver tissue fixed in 4% paraformaldehyde was embedded in paraffin, sectioned into 5 *μ*m thickness, and stained with hematoxylin-eosin (H&E) for evaluation of histopathological changes. The histopathological changes of stained liver slices were observed under a bright-field microscope.

### 2.8. Ferric-Reducing Antioxidant Power (FRAP) Assay

The FRAP assay was performed based on the method described in the literature [9]. In brief, the FRAP reagent was prepared from 10 mmol/L TPTZ solution, 20 mmol/L iron(III) chloride solution, and 300 mmol/L sodium acetate buffer solution (pH 3.6) in a volume ratio of 1 : 1 : 10, respectively. 100 *μ*L of the diluted sample was added to 3 mL of the FRAP reagent and the mixture was measured after 4 min at 593 nm. The standard curve was established using FeSO_4_ solution, and the results were expressed as *μ*mol Fe(II)/g dry weight of lemon.

### 2.9. Trolox Equivalent Antioxidant Capacity (TEAC) Assay

The TEAC assay was carried out according to the procedure in the literature [10]. Briefly, the ABTS^•+^ stock solution was prepared from 2.45 mmol/L potassium persulfate and 7 mmol/L ABTS solution in a volume ratio of 1 : 1 and then placed in the dark for 16 h at room temperature. The ABTS^•+^ working solution was prepared by diluting the stock solution, and the absorbance of ABTS^•+^ working solution was 0.710 ± 0.05 at 734 nm. 100 *μ*L of the diluted sample was mixed with 3.8 mL ABTS^•+^ working solution, and the absorbance of the mixture was measured at 734 nm after 6 min, and the percent of inhibition of absorbance at 734 nm was calculated. The reference standard was Trolox, and the results were expressed as *μ*mol Trolox/g dry weight of lemon.

### 2.10. Determination of TPC

TPC were measured according to the literature [11]. Briefly, 0.50 mL of the diluted sample was added to 2.5 mL of 0.2 mmol/L Folin–Ciocalteu reagent. After 4 min, 2 mL of saturated sodium carbonate solution was added. After incubation for 2 h at room temperature, the absorbance of the mixture was measured at 760 nm. The reference standard was gallic acid, and the results were expressed as mg gallic acid equivalent (GAE)/g dry weight of lemon.

### 2.11. Determination of TFC

TFC were measured according to the literature [12]. In brief, 0.50 mL of the sample was mixed with 1.5 mL of 95% ethanol (v/v), 0.1 mL of 10% aluminum chloride (w/v), 0.1 mL of 1 mol/L potassium acetate, and 2.8 mL of water. After incubation for 30 min at room temperature, the absorbance of the mixture was determined at 415 nm. The reference standard was quercetin, and the results were expressed as mg of quercetin equivalent (QE)/g dry weight of lemon.

### 2.12. Statistical Analysis

Statistical analysis was carried out by one-way analysis of variance (ANOVA) with post hoc LSD test using SPSS 13.0 software. *p* < 0.05 was regarded as significant.

## 3. Results

### 3.1. Effects of Lemon Juice on the Levels of ALT and AST in Serum

**Figure 1:**
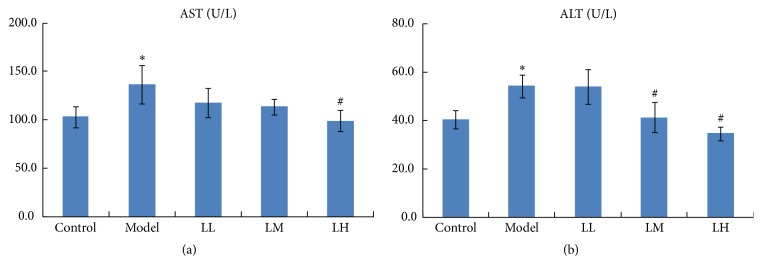
Effects of lemon juice on the levels of AST (a) and ALT (b) in serum of mice. Control: normal group; Model: alcohol group; LL: alcohol and low dose of lemon juice group; LM: alcohol and medium dose of lemon juice group; LH: alcohol and high dose of lemon juice group. *∗* means the levels of parameters in the model group were significantly (*p* < 0.05) different from those of the control group. # means the levels of parameters in the treatment group were significantly (*p* < 0.05) different from those of the model group.

As shown in Figure 1, the administration of alcohol led to a significant (*p* < 0.05) elevation of alanine transaminase (ALT) and aspartate transaminase (AST) levels in serum of the model group compared with that of the control group. The administration of low and medium concentration of lemon juice slightly prevented the elevation of serum level of AST, while a high dose of lemon juice significantly (*p* < 0.05) decreased it. At the same time, the prevention of the elevation of serum levels of ALT was observed significantly (*p* < 0.05) in medium and high concentration of lemon juice group and displayed a dose-effect relationship.

### 3.2. Effects of Lemon Juice on the Levels of TG in Serum and Liver

**Figure 2:**
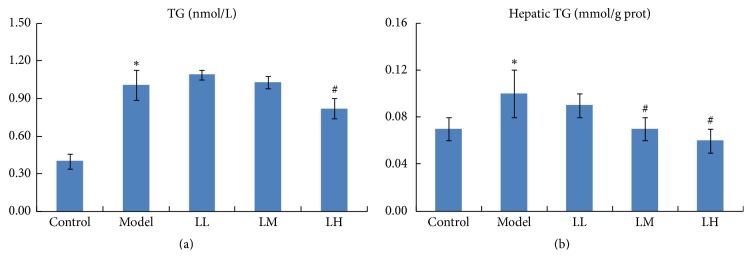
Effects of lemon juice on TG contents in serum (a) and liver (b). Control: normal group; Model: alcohol group; LL: alcohol and low dose of lemon juice group; LM: alcohol and medium dose of lemon juice group; LH: alcohol and high dose of lemon juice group. *∗* means the levels of parameters in the model group were significantly (*p* < 0.05) different from those of the control group. # means the levels of the parameters in the treatment group were significantly (*p* < 0.05) different from those of the model group.

Triacylglycerol (TG) content in serum was significantly (*p* < 0.05) increased in the model group compared with that in the control group (Figure 2(a)). Administration of lemon juice reduced the accumulation of TG in a dose-dependent manner, especially in high concentration of lemon juice group (*p* < 0.05). In addition, hepatic TG content was significantly (*p* < 0.05) increased in model group compared with that in the control group (Figure 2(b)). Administration of medium and high concentration of lemon juice significantly (*p* < 0.05) reduced the accumulation of hepatic TG in a dose-dependent manner.

### 3.3. Effects of Lemon Juice on Liver Lipid Peroxidation Levels

**Figure 3:**
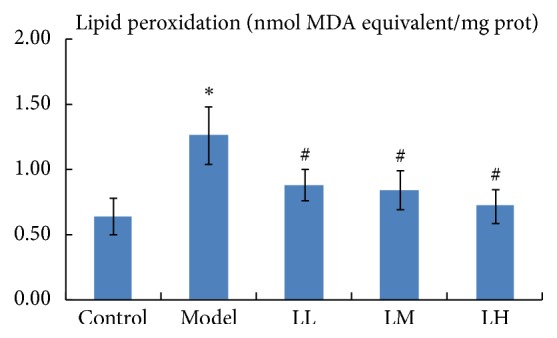
Effects of lemon juice on hepatic lipid peroxidation level in mice. Control: normal group; Model: alcohol group; LL: alcohol and low dose of lemon juice group; LM: alcohol and medium dose of lemon juice group; LH: alcohol and high dose of lemon juice group. *∗* means the levels of the parameters in the model group were significantly (*p* < 0.05) different from those of the control group. # means the levels of the parameters in the treatment group were significantly (*p* < 0.05) different from those of the model group.

The lipid peroxidation levels in liver tissue are shown in Figure 3. Compared with that of the control group, there was a significant (*p* < 0.05) increase in the lipid peroxidation level of the model group. The administration of lemon juice significantly (*p* < 0.05) decreased the level of lipid peroxidation in a dose-dependent manner.

### 3.4. Effects of Lemon Juice on Liver Antioxidant Enzyme Activities

**Figure 4:**
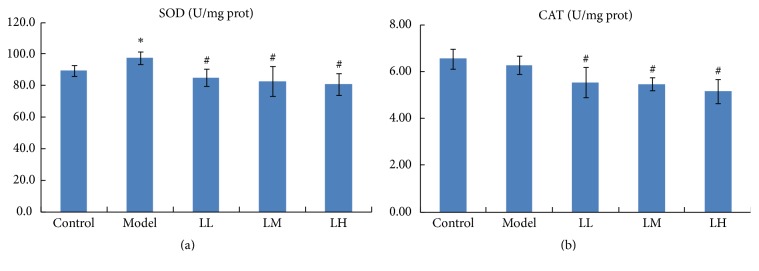
Effects of lemon juice on the activities of SOD (a) and CAT (b) in liver. Control: normal group; Model: alcohol group; LL: alcohol and low dose of lemon juice group; LM: alcohol and medium dose of lemon juice group; LH: alcohol and high dose of lemon juice group. *∗* means the levels of the parameters in the model group were significantly (*p* < 0.05) different from those of the control group. # means the levels of the parameters in the treatment group were significantly (*p* < 0.05) different from those of the model group.

Figure 4 represents the results of hepatic antioxidant enzyme activities in five groups. The SOD level in the liver increased significantly (*p* < 0.05) in the model group compared with that in the control group. The CAT level in the liver decreased only slightly (*p* > 0.05) in the model group compared with the control group in this study. However, treatment with lemon juice significantly (*p* < 0.05) decreased the levels of SOD and CAT compared with those of the model group. In addition, all the biochemical parameters are summarized in Table 1.

### 3.5. Histopathological Evaluation

**Figure 5:**
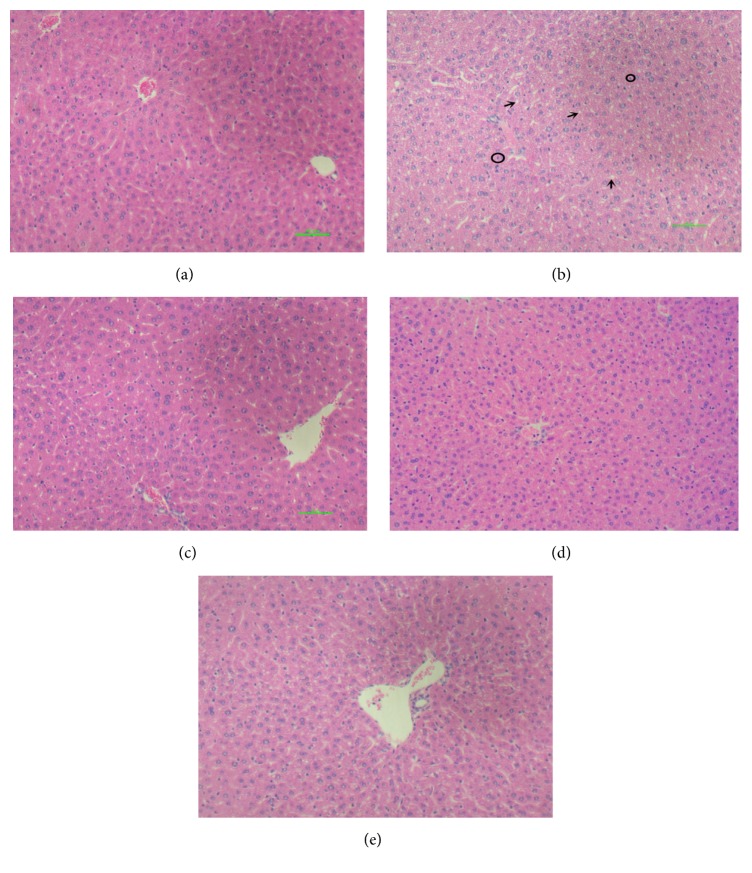
The photomicrographs of liver sections taken from mice. (a) Normal group; (b) alcohol group; (c) alcohol and low dose of lemon juice group; (d) alcohol and medium dose of lemon juice group; (e) alcohol and high dose of lemon juice group. Arrow indicates a condition of small fat droplets changes, and the circle indicates hepatocytes necrosis, which mainly occurs in alcohol model group.

Histopathology assessment of the liver was carried out for all groups (Figure 5). There was no pathological abnormality observed in the liver of the control group with preserved cytoplasm and distinct nucleus as shown in Figure 5(a). In Figure 5(b), it was observed in the model group that ethanol induced necrosis changes and substantial small fat droplets changes in liver section. However, livers of mice in all lemon juice treated groups showed noticeable recovery from ethanol induced liver damage with fewer small fat droplets changes and hepatocytes necrosis features.

### 3.6. The In Vitro Antioxidant Activity, Total Phenolic Contents (TPC), and Total Flavonoid Contents (TFC) of Lemon Juice

The in vitro antioxidant activities of lemon were evaluated using ferric-reducing antioxidant power (FRAP) and Trolox equivalent antioxidant capacity (TEAC) assays. The FRAP and TEAC values were 50.82 ± 2.70 *μ*mol Fe(II)/g dry weight (DW) and 19.88 ± 0.66 *μ*mol Trolox/g DW, respectively. The total phenolic contents (TPC) and total flavonoid contents (TFC) of lemon were 6.21 ± 0.28 mg GAE/g DW and 0.30 ± 0.03 mg QE/g DW, respectively.

## 4. Discussion

Alcohol use disorder causes substantial diseases, and the liver is the most adversely affected organ. In the present study, the effects of lemon juice on chronic alcohol-induced liver injury in mice were investigated. Ethanol induced impairment of liver in mice was evidenced by increased AST and ALT levels. Treatment with lemon juice lowered the increased levels of AST and ALT in serum. The return of the activities of aminotransferases (AST or ALT) in serum to normal indicates the regeneration of hepatocytes and the healing of hepatic parenchyma; therefore, lemon juice had a protective effect on alcohol-induced liver injury. The results were in agreement with previous reports that showed lemon possessing a hepatoprotective effect on liver injury induced by carbon tetrachloride and acute exercise [7, 13]. In addition, the chronic alcohol-induced liver damage was further confirmed by liver histopathological changes in the present study, and treatment with lemon juice also remarkably improved the liver histopathological changes, which further confirmed the hepatoprotective activity of lemon juice on alcohol-induced liver injury in mice.

Various factors and mechanisms are associated with the pathological progress of alcohol-induced liver injury, and oxidative stress was one of them [3]. ROS is one kind of prooxidants including hydroxyl radical, superoxide radical, and hydrogen peroxide, which are frequently generated spontaneously during metabolism. Normally produced ROS is rapidly eliminated by the antioxidant defense system. The antioxidant defense system is able to scavenge ROS and terminate chain reaction of free radicals in vivo. Alcoholic exposure can result in excessive accumulation of ROS and contribute to cellular damage. Excessive accumulation of ROS could cause lipid peroxidation of hepatocytes, which was regarded as the primary mechanism concerned with chronic alcohol-induced liver damage [8]. MDA, the product of lipid peroxidation induced by ROS, also accumulates in the alcohol-damaged liver and represents a good estimation of the total oxidative stress [3]. In the present study, alcohol significantly augmented lipid peroxidation levels, which was similar to the previous study that showed increased lipid peroxidation in alcoholic patients [14]. Treatment with lemon juice reduced the level of lipid peroxidation to a normal level, which showed a significant protective effect of lemon juice against alcohol-induced oxidative stress.

Liver steatosis is the earliest disease of the liver on account of chronic ethanol consumption, with the characteristic of fat accumulation. It is generally accepted that, in the development of hepatic steatosis, ethanol exposure increases the ratio of reduced nicotinamide adenine dinucleotide/oxidized nicotinamide adenine dinucleotide in hepatocytes, which disturb mitochondrial fatty acid *β*-oxidation and induce steatosis further [15]. In this study, alcohol-induced occurrence of hepatic steatosis was confirmed by increased hepatic TG contents and histopathological changes. Treatment with lemon juice significantly lowered the hepatic TG contents and improved the damaged histopathological changes. In particular, the mice given high dose of lemon juice had almost completely recovered to normal.

The antioxidant enzymes, such as SOD and CAT, represent the defense response system to excessive ROS. SOD catalyzes the dismutation of two superoxide anions to hydrogen peroxide and oxygen, and then CAT degrades two hydrogen peroxide molecules to water and oxygen [16]. SOD is also considered as front line among antioxidant enzymes in defense against free radicals. In the literature, the effects of alcohol treatment on the levels of SOD/CAT are controversial. SOD showed an increase, no changes, or a decrease, depending on the model, diet, duration, and amount of alcohol consumption [17–19]. In addition, it was reported that CAT activity decreased upon chronic ethanol consumption in a study [20]. However, another study showed that CAT activity was increased in rat liver [18]. In our study, the alcohol treatment significantly increased the activity of SOD and slightly decreased the activity of CAT, while treatment with lemon juice decreased the activities of SOD and CAT. The increased activity of SOD reflects the activation of the compensatory mechanism which might be an attempt to counteract free radicals in the liver [21]. The treatment with lemon juice prevented ROS accumulation, and the compensatory effects were not available in the liver. Therefore, lemon juice decreased the activities of SOD and CAT. The results were similar to the report of Gasparotto et al. [22]. In addition, the in vitro antioxidant experiment of lemon also showed that lemon had medium in vitro antioxidant capacities, which contribute to the explanation of the in vivo free radical scavenging effect of lemon.

Lemon contains numerous beneficial bioactive compositions, including phenolic compounds (mainly flavonoids), vitamins, carotenoids, essential oils, minerals, and dietary fiber [6]. The hepatoprotective effect of lemon may be attributable to the presence of vitamins, flavonoids, essential oils, and pectin. Vitamin C, a water-soluble antioxidant in lemon, is in a unique position to scavenge aqueous peroxyl radicals and react with free radicals, thus preventing oxidative damage including lipid peroxidation [14]. Sometimes, vitamin C could exert prooxidative effects at low concentrations and in the existence of transition metal ions [23], which might aggravate oxidative stress. However, it is difficult for vitamin C to have prooxidative effects in vivo due to the presence of NADPH-dependent recycling systems and glutathione [24]. In addition, there were some literatures reporting that vitamin C supplementation alone could reduce oxidative stress induced by ethanol, and the hepatoprotective effect of vitamin C treatment was more effective than silymarin, quercetin, and thiamine [25, 26]. Flavonoids, a class of secondary plant phenolics, can interact with hydroxyl radicals, chelate metal catalysts, and inhibit oxidases [27]. In previous studies, lemon flavonoid was shown to possess a hepatoprotective effect on liver damage induced by carbon tetrachloride and acute exercise, and the mechanism of the protective effect was related to the antioxidant capacity [7, 13]. Lemon essential oils and pectin were found to have protective effects on stomach and intestine barrier function [28, 29]. Ethanol exposure can injure the defensive intestinal barrier and increase the permeability of the small intestine, which lead to bacterial endotoxins leakage [25]. The bacterial endotoxins leakage is an important factor in the pathogenesis of alcohol-induced liver injury [30]. Therefore, the lemon essential oils and pectin might protect the intestine barrier function, thus indirectly protecting against alcohol-induced liver injury.

In this study, lemon juice revealed a protective effect on chronic alcohol-induced liver injury. Due to the fact that lemon contains a variety of bioactive ingredients, the hepatoprotective effect might be the result of joint action of multiple mechanisms, and it is difficult to clarify the specific mechanism of effect. The medium in vitro antioxidant capacities of lemon and reduced in vivo MDA levels indicated that lemon might reduce the oxidative stress induced by ethanol, thus exerting hepatoprotective effects. This study has found that lemon juice has a strong hepatoprotective effect, which provides valuable information for the general public to reduce harm of alcohol consumption. In the future, active components in lemon juice should be separated and identified, and the mechanism of action of the purified compound should be explored, including the action on the small intestine.

## 5. Conclusions

Chronic alcohol consumption could induce liver injury. Lemon juice is readily available as a widely consumed beverage. In this study, we found that treatment with lemon juice exerted hepatoprotective effects on alcohol-induced liver injury in mice through decreasing the levels of serum ALT and AST as well as hepatic TG and lipid peroxidation. In addition, the in vitro antioxidant experiment of lemon showed that lemon had medium in vitro antioxidant capacities. Therefore, we speculate that the hepatoprotective effects might be related to the antioxidant capacities of lemon juice. The results showed that lemon juice might be a potential dietary supplement for the prevention and treatment of liver injury related to chronic alcohol consumption.

## Figures and Tables

**Table 1:** Effects of lemon juice on the levels of several biochemical parameters.

Parameters	Control	Model	LL	LM	LH
AST (U/L)	103 ± 10.45	136.53 ± 19.94^*∗*^	117.88 ± 15.37	113.5 ± 7.7	98.85 ± 10.94^#^
ALT (U/L)	40.5 ± 3.89	54.32 ± 4.76^*∗*^	54.05 ± 7.18	41.32 ± 6.25^#^	34.68 ± 2.71^#^
Serum TG (nmol/L)	0.4 ± 0.06	1.01 ± 0.12^*∗*^	1.09 ± 0.04	1.03 ± 0.05	0.82 ± 0.08^#^
Liver TG (mmol/g prot)	0.07 ± 0.01	0.1 ± 0.02^*∗*^	0.09 ± 0.01	0.07 ± 0.01^#^	0.06 ± 0.01^#^
Lipid peroxidation (nmol MDA equivalent/mg prot)	0.64 ± 0.14	1.26 ± 0.22^*∗*^	0.88 ± 0.12^#^	0.84 ± 0.15^#^	0.72 ± 0.13^#^
SOD (U/mg prot)	89.6 ± 3.42	97.51 ± 3.96^*∗*^	85.27 ± 5.57^#^	83 ± 9.28^#^	81.03 ± 6.65^#^
CAT (U/mg prot)	6.55 ± 0.41	6.29 ± 0.39	5.55 ± 0.64^#^	5.47 ± 0.28^#^	5.17 ± 0.51^#^

*Note*. Control: normal group; Model: alcohol group; LL: alcohol and low dose of lemon juice group; LM: alcohol and medium dose of lemon juice group; LH: alcohol and high dose of lemon juice group. *∗* means the levels of the parameters in the model group were significantly (*p* < 0.05) different from that of the control group. # means the levels of the parameters in the treatment group were significantly (*p* < 0.05) different from that of the model group.
